# Anthropometry and Body Composition in Childhood: Follow‐Up of a Randomised, Double‐Blinded Controlled Trial With a Modified, Low‐Protein Infant Formula During Infancy

**DOI:** 10.1111/ijpo.70038

**Published:** 2025-07-04

**Authors:** Jacqueline Muts, Stefanie M. P. Kouwenhoven, Nadja Antl, Marieke Abrahamse‐Berkeveld, Britt J. van Keulen, Jos W. R. Twisk, Dewi van Harskamp, Chris H. P. van den Akker, Berthold Koletzko, Johannes B. van Goudoever

**Affiliations:** ^1^ Department of Pediatrics, Emma Children's Hospital, Amsterdam UMC University of Amsterdam Amsterdam the Netherlands; ^2^ Amsterdam Reproduction & Development Research Institute, Amsterdam UMC Amsterdam the Netherlands; ^3^ Erasmus MC‐Sophia, Department of Pediatric & Neonatal Intensive Care Rotterdam the Netherlands; ^4^ Department of Internal Medicine, Division of Dietetics Erasmus MC University Medical Centre Rotterdam Rotterdam the Netherlands; ^5^ Department of Pediatrics, Dr. von Hauner Children's Hospital, LMU Hospital, LMU – Ludwig‐Maximilians‐Universität Munich Munich Germany; ^6^ German Center for Child and Adolescent Health, Site Munich Germany; ^7^ Danone Research & Innovation Utrecht the Netherlands; ^8^ Department of Epidemiology and Data Science Amsterdam University Medical Center Amsterdam the Netherlands; ^9^ Amsterdam UMC, Department of Laboratory Medicine, Core Facility Metabolomics, Laboratory Genetic Metabolic Disease University of Amsterdam Amsterdam the Netherlands; ^10^ Amsterdam UMC, Emma Center for Personalized Medicine Amsterdam the Netherlands; ^11^ Amsterdam UMC, Amsterdam Gastroenterology, Endocrinology & Metabolism Research Institute, Amsterdam UMC Amsterdam the Netherlands; ^12^ Department of Neonatology, Emma Children's Hospital, Amsterdam UMC University of Amsterdam Amsterdam the Netherlands

**Keywords:** air‐displacement plethysmography, amino acids, early childhood, infant formula, obesity, protein intake

## Abstract

**Introduction:**

Formula feeding is associated with an increased obesity risk compared to breastfeeding, possibly due to its higher protein content. We aimed to investigate the influence of feeding a modified, low‐protein infant formula during the first 6 months of life on growth and body composition at 6 years.

**Methods:**

Healthy term‐born infants were randomised to receive a low‐protein (mLP) infant formula with modified amino acid composition (*n* = 90; 1.7 g protein/100 kcal) or a control infant formula (CTRL) (*n* = 88; 2.1 g protein/100 kcal) up to 6 months. A breastfed (BF, *n* = 67) group served as a reference. At 6 years, anthropometry and body composition (air‐displacement plethysmography) were measured.

**Results:**

A total of 106 infants were measured at 6y follow‐up, (*n* = 39 mLP; *n* = 33 CTRL; *n* = 34 BF). No significant differences were observed in mean weight, length, BMI, or fat mass percentage. However, the mean fat‐free mass was lower in the mLP compared to the CTRL group (−1240 g; 95% CI: −1889 to −591, *p <* 0.001). Moreover, both formula groups had a higher absolute fat mass when compared to the breastfed group (*p* = 0.01).

**Conclusions:**

Feeding a mLP formula during early life did not have beneficial effects on body composition in a subset of infants studied at age 6 year.

**Abbreviations**
ADP: air‐displacement plethysmography
BF: breastfed
CI: confidence interval
CTRL: control
D_2_O: deuterium oxide
FFM: fat‐free mass
FFMI: fat‐free mass index
FM: fat mass
FM%: fat mass percentage
FMI: fat mass index
LMM: linear mixed model
mLP: modified low‐protein
SDS: standard deviation score
TBW: total body water

## 1 Introduction

Childhood obesity is a major global health concern, with its prevalence rising over the past decades. The World Health Organisation reported that approximately 37 million children under the age of five were living with overweight in 2022 [1]. Childhood obesity often persists into adulthood [2, 3] and is associated with obesity‐related morbidities in adulthood, such as diabetes and cardiovascular diseases [4]. Multiple studies have shown that early nutritional interventions can have effects on long‐term health outcomes, including obesity risk, indicating that early life nutrition plays a crucial role in programming metabolic and physiological processes that may persist into adulthood [5, 6, 7, 8]. During this critical period, infants who receive infant formula often have a higher weight gain and more adipose tissue compared to infants who are breastfed [9, 10]. Thus, breastfeeding results in a more optimal body composition compared to feeding conventional infant formula [11, 12, 13].

One of the differences between infant formula and breastmilk is that formula typically contains a higher protein content than breastmilk, with one reason being the aim to safeguard an adequate intake of essential amino acids [14, 15]. The so‐called ‘early protein hypothesis’ states that a higher protein intake during early life leads to an increased weight gain and adipose tissue gain in the first few years of life [16]. A large well‐designed study has examined the differences between lower protein (1.77 g protein/100 kcal, and 2.2 g protein/100 kcal follow‐on formula) and higher protein (2.9 g protein/100 kcal, and 4.4 g protein/100 kcal follow‐on formula) formulas, finding that infants fed higher protein formulas experience accelerated growth rates and fat mass development [17], although another high quality study (1.8 versus 2.7 g/100 kcal) with smaller sample size did not confirm this observation [18]. This may suggest that reducing the protein content in infant formula may lead to a “healthier” body composition (i.e., one that mimics the body composition pattern of breastfed infants) in early childhood and potentially later in life. However, the optimal amount of protein or to which extent the protein content can be lowered by optimising protein composition while still safeguarding adequate infant growth remains unknown. Previously, amino acid requirement assessments of formula fed infants were conducted aimed to optimise the composition of infant formulas [19, 20, 21, 22, 23, 24].

Previously we have shown in our randomised controlled trial (RCT), called ProtEUs, that, compared to a conventional term infant formula (control; CTRL) (2.1 g protein/100 kcal), feeding a new modified lower‐protein infant formula (mLP) (1.7 g protein/100 kcal with an optimised amino acid profile) during the first 6 months of life is safe and supports adequate growth without differences in body composition during the first 2 years of life [25, 26]. We continued our ProtEUs study to investigate the potential long‐term effects of the aforementioned low‐protein infant formula on growth and body composition at 6 years of age.

## 2 Methods

### 2.1 Original Trial and Follow‐Up at 1 and 2 Years

During the original ProtEUs study [25], healthy term‐born formula‐fed infants were randomised to exclusively receive one of two non‐commercial formulas: either a mLP infant formula (1.7 g protein/100 kcal) or an isocaloric CTRL infant formula (2.1 g protein/100 kcal) up to the age of 6 months [25]. Apart from the differences in protein levels, the infant formulas differed in amino acid composition, and both comprised 70% intact protein and 30% free amino acids. The control formula was nearly identical in composition to the standard commercial infant formula from Nutricia except for the partial amino acid content. A group of breastfed (BF) healthy term‐born infants was included as a reference group. All infants were enrolled within the first 45 days postnatally. Between 2014 and 2016, a total of 245 infants were enrolled in the study, of whom 178 were randomly assigned to receive either the mLP or CTRL formula. Subsequently, both groups received a standard follow‐on formula up to the age of 1 year. A total of 67 infants were included in the BF reference group. The reference group was exclusively breastfed, with the exception of one standard formula feed per day allowed. Complementary feeding, across all three feeding groups, was introduced no earlier than 17 weeks of age, in accordance with national guidelines.

Both parents, caregivers, and researchers were blinded to group allocation. The intervention period of 6 months was followed by two follow‐up visits at the ages of one and 2 years. During these visits, safety parameters, growth, and body composition were measured. A more detailed description of the intervention period and study procedures up to the age of 2 years has been described previously, as well as the (amino acid) composition of both intervention formulas used in the trial (Table S1) [25, 26].

The study was conducted at Amsterdam UMC, VU University Medical Center, Amsterdam, The Netherlands and the Dr. von Hauner Children's Hospital, Ludwig‐Maximilians‐Universität, Munich, Germany. The trial was approved by the medical ethical review boards of VU University Medical Center Amsterdam and the medical faculty of LMU Munich. This trial was registered in the Dutch Trial Register (Study ID number NTR4829, trial number NL4677). The study was conducted according to ICH‐GCP and in compliance with the Declaration of Helsinki.

Written informed consent was obtained from all participants' parents or guardians, both for the trial period and the follow‐up period.

### 2.2 Design and Outcomes of Follow‐Up Study at the Age of 6 Years

For the current study, a pre‐planned visit was scheduled at the age of 6 years. During this visit, anthropometry and body composition were assessed. Weight was measured on a balance scale (MS‐4100, MARSDEN, UK) to 0.5 g accuracy, height was measured using a digital stadiometer (SECA 285, UK), and head circumference was measured using a non‐stretchable tape measure, both to 0.1 cm accuracy.

Body composition was measured with air‐displacement plethysmography (ADP) (BOD POD Body Composition System; Cosmed, Concord, CA, USA). With the generated fat mass (FM) and fat‐free mass (FFM), the fat mass index (FMI) and fat‐free mass index (FFMI) were calculated with the following equations: FM (kg)/length^2^ (m) and FFM (kg)/length^2^ (m), respectively [27].

The triceps, biceps, subscapular, and suprailiac skinfold thicknesses were measured using a Holtain Skinfold Calliper (Holtain LTD., Crosswell, UK) to an accuracy of 0.1 mm.

An additional method for assessing body composition involves the deuterium dilution technique. Initially, a baseline saliva sample (t0) is collected, followed by the oral administration of 0.3 mL/Kg body weight of deuterium oxide (D_2_O). Subsequent saliva samples are collected at five specific time points within a week (after 6, 24, 48, 96, and 168 h) with an oracol + collection system (Malvern Medical Developments, Worcester, United Kingdom) and stored frozen at −20°C for later analysis. The saliva is recovered from the collection systems by centrifugation (10 min, 1800 × g, 20°C), and deuterium enrichments are analysed using High Temperature Conversion Elemental Analyser coupled to Isotope Ratio Mass Spectrometry (TC/EA‐IRMS, Thermo Scientific, Bremen, Germany). Total body water (TBW) is determined from the results through the back extrapolation method. This method involves plotting the logarithm of isotopic enrichment against time to estimate deuterium enrichment at the time of dosing. TBW is determined by the formula: TBW = dose (mol) / enrichment at extrapolated time of dosing [28, 29]. FFM is then calculated from TBW using age‐ and sex‐specific conversion factors for children up to 10 years of age [30]. FM is derived as the difference between body weight and FFM, and FM percentage (FM%) is calculated using the formula: FM% = [(body mass—FFM)/body mass] × 100%.

### 2.3 Statistical Analyses

Categorical variables are reported as frequencies (%) and continuous parametric variables are expressed as mean values and their standard deviations (SDs), provided the data were distributed parametrically.

Differences in anthropometry and body composition between feeding groups at 6 years of age were analysed with linear mixed models (LMMs) and expressed as absolute differences along with their 95% confidence intervals (CIs). LMMs help deal with longitudinal data by correlating measurements at different times within a subject via a random intercept at the subject level. This approach allows all group comparisons (mLP vs. CTRL, mLP vs. BF, and CTRL vs. BF) to be obtained from the same model by changing the reference category in the group variable. Additionally, LMMs handle missing data by including all available data from the repeated measures, even from participants who did not complete the full 6‐year follow‐up. The analyses were adjusted for sex, birth weight standard deviation scores (SDS) (WHO Child Growth Standards [31]), baseline measurement and age at visit. We included these co‐variates, as sex and birth weight are known to have a correlation with weight and body composition at later stages [32, 33]. Moreover, age at visit was included to account for potential variations between study subjects ages, which could deviate by several months from the designated 6‐year mark.

Statistical analyses were performed using IBM SPSS version 28 for Windows [34]. Two‐sided statistical significance was assumed at *p*‐values < 0.05.

## 3 Results

**FIGURE 1:**
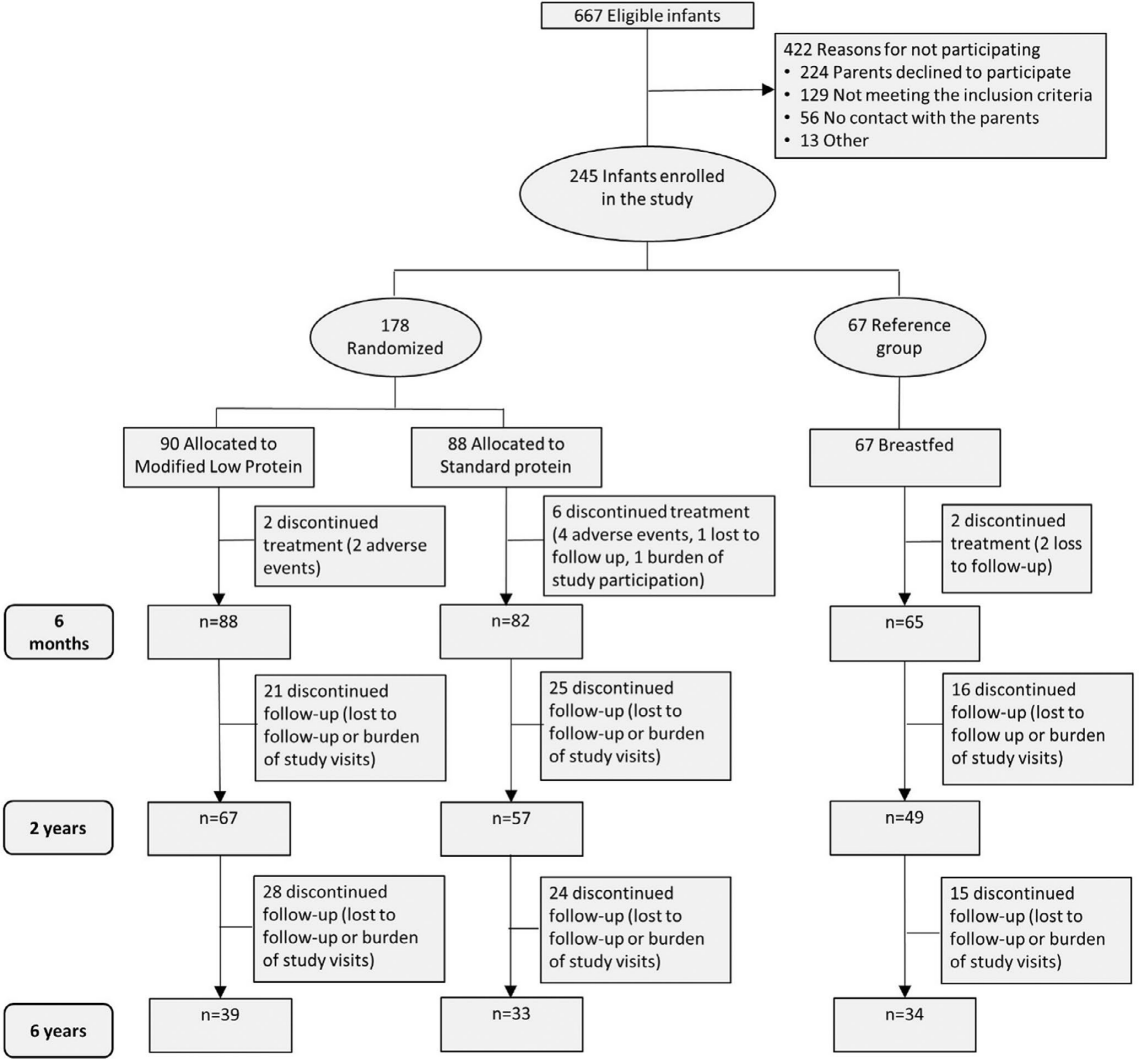
Flow‐chart diagram of progression of participants through the study.

In the original intervention study, a total of 245 infants were enrolled. At the final follow‐up invitation at 6 years of age, COVID‐19 pandemic restrictions were applicable so that only 106 children (43%) were able to visit the out‐patient clinic for anthropometry (Figure 1). Of these participants, 90 children had a successful body composition measurement with ADP.

In this bicentric study, 58% of the measurements at 6‐year follow‐up were conducted at Amsterdam UMC and 42% at Dr. von Hauner Children's Hospital Munich.

All baseline infant and maternal characteristics were similar in the three feeding groups of this follow‐up population (Table 1). The mean age of infants included at the follow‐up was 6 years and 9 months, somewhat beyond the planned age of 6 years, also mainly due to COVID‐19 restrictions during the planned study period.

**TABLE 1:** Baseline characteristics of follow‐up population in each feeding group at 6 years of age (*n* = 106).

	mLP (*n* = 39)	CTRL (*n* = 33)	BF (*n* = 34)
Age at follow up 6 years (months)	80.1 ± 8.8	80.8 ± 8.6	82.5 ± 8.3
Male sex (%)	17 (44)	15 (46)	19 (53)
Birth weight (kg)	3.36 ± 0.34	3.35 ± 0.35	3.51 ± 0.30
Birth weight (SDS)	0.14 ± 0.73	0.13 ± 0.73	0.43 ± 0.61
Caucasian (%)	33 (85)	31 (94)	32 (89)
Gestational age (weeks)	39.4 ± 1.2	39.6 ± 1.2	39.7 ± 0.95
Maternal BMI at study entry (kg/m^2^)	24.1 ± 3.7	26.3 ± 3.5	23.2 ± 3.5

*Note*: Values presented as mean ± SD or *n* (%). *p* value tested with ANOVA for continuous variables and Chi‐square test for categorical variables comparing all three feeding groups.

Abbreviations: BF, breastfed; CTRL, control; mLP, modified low protein.

The baseline characteristics of the subgroup of participants who took part in the 6‐year follow‐up were similar to those of the entire sample during the intervention period (Table S2). Therefore, the follow‐up subgroup is representative of the overall sample.

### 3.1 Anthropometric Outcomes

No significant differences between any of the groups were observed in anthropometric outcomes, including weight, height, BMI, skinfold thicknesses, and head circumference, at 6 years of age (Tables 2 and 3; Figure S1A–C).

**TABLE 2:** Anthropometric data in each feeding group at 6 years of age (*n* = 106) and the differences between the feeding groups.

Anthropometric data	mLP	CTRL	BF	mLP minus CTRL		mLP minus BF		CTRL minus BF	
	(*n* = 39)	(*n* = 33)	(*n* = 34)	Diff (95% CI)	*p*	Diff (95% CI)	*p*	Diff (95% CI)	*p*
Weight (kg)	22.6 ± 3.3	23.2 ± 3.8	22.8 ± 3.1	−0.48 (−1.3–0.37)	0.27	0.17 (−0.16–0.49)	0.32	0.16 (−0.18–0.50)	0.35
Length (cm)	122 ± 5.4	123 ± 5.8	122 ± 6.0	−1.01 (−2.4–0.30)	0.13	0.18 (−1.17–1.53)	0.79	1.25 (−0.17–2.7)	0.08
BMI (kg/m^2^)	15.2 ± 1.7	14.9 ± 3.0	15.2 ± 1.1	0.31 (−0.37–1.0)	0.37	0.25 (−0.44–0.93)	0.48	−0.07 (−0.78–0.64)	0.85
Head circumference (cm)	51.6 ± 2.4	51.7 ± 1.3	52.0 ± 1.8	−0.07 (−0.71–0.56)	0.82	−0.06 (−0.70–0.58)	0.85	0.01 (−0.64–0.67)	0.97

*Note*: Values presented as mean ± SD. Differences tested with Linear mixed models. Adjusted for gender, birth weight SDS, age at visit and baseline value of outcome variable.

Abbreviations: BF, breastfed; CTRL, control; mLP, modified low protein.

**TABLE 3:** Body composition measured with air‐displacement plethysmography (ADP) (*n* = 90), skinfold thickness (*n* = 106) and body composition measured with D_2_O Dilution Technique (*n* = 27) in each feeding group at 6 years of age and differences between the feeding groups.

ADP	mLP	CTRL	BF	mLP minus CTRL		mLP minus BF		CTRL minus BF	
	(*n* = 30)	(*n* = 29)	(*n* = 31)	Diff (95% CI)	*p*	Diff (95% CI)	*p*	Diff (95% CI)	*p*
Fat mass (kg)	3.5 ± 1.1	3.6 ± 1.7	3.2 ± 1.7	−0.02 (−0.45–0.42)	0.95	0.38 (0.11–0.81)	**0.01**	0.39 (0.10–0.83)	**0.01**
Fat mass percentage (%)	14.1 ± 5.4	14.2 ± 6.1	13.3 ± 6.0	0.40 (−2.2–3.0)	0.76	1.2 (−1.4–3.7)	0.38	0.76 (−1.8–3.4)	0.57
Fat‐free mass (kg)	19.0 ± 1.9	20.0 ± 3.3	19.6 ± 2.3	−0.97 (−1.6 – −0.39)	**0.001**	−0.39 (−0.98–0.20)	0.19	0.58 (−0.01–1.2)	0.06
FMI (kg/m^2^)	2.3 ± 0.69	2.3 ± 1.1	2.1 ± 0.99	0.15 (−0.32–0.60)	0.54	0.36 (−0.10–0.82)	0.13	0.22 (−0.25–0.68)	0.37
FFMI (kg/m^2^)	12.8 ± 1.1	12.8 ± 2.6	13.1 ± 0.77	0.12 (−0.38–0.62)	0.63	−0.06 (−0.57–0.45)	0.81	−1.18 (−0.70–0.33)	0.48

Skinfold thickness	(*n* = 39)	(*n* = 33)	(*n* = 34)	Diff (95% CI)	*p*	Diff (95% CI)	*p*	Diff (95% CI)	*p*
Triceps right (mm)	9.1 ± 2.2	9.6 ± 2.4	8.7 ± 2.5	−0.41 (−1.3–0.53)	0.39	0.61 (−0.32–1.5)	0.20	1.02 (−0.05–1.98)	0.06
Triceps left (mm)	9.4 ± 2.2	9.3 ± 2.1	9.1 ± 2.5	0.10 (−0.81–1.01)	0.83	0.47 (−0.43–1.37)	0.30	0.37 (−0.56–1.3)	0.44
Biceps right (mm)	5.8 ± 1.8	6.3 ± 2.2	5.7 ± 2.1	−0.55 (−1.3–0.16)	0.13	0.21 (−0.50–0.91)	0.57	0.75 (−0.02–1.48)	0.06
Biceps left (mm)	5.8 ± 2.0	6.0 ± 1.8	5.7 ± 2.5	−0.14 (−0.86–0.58)	0.70	0.14 (−0.58–0.85)	0.71	0.28 (−0.46–1.02)	0.46
Subscapular (mm)	5.5 ± 2.0	5.5 ± 1.3	5.6 ± 1.4	0.02 (−0.67–0.71)	0.96	−0.06 (−0.75–0.63)	0.86	−0.08 (−0.79–0.63)	0.83
Suprailiac (mm)	6.1 ± 2.2	6.1 ± 2.7	6.2 ± 2.9	−0.25 (−0.95–0.90)	0.96	−0.11 (−1.0–0.81)	0.81	−0.09 (−1.0–0.86)	0.86
Summed (mm)	28.2 ± 5.3	29.2 ± 5.5	27.2 ± 6.0	−1.20 (−3.84–1.45)	0.38	−0.03 (−2.6–2.6)	0.98	1.17 (−1.5–3.8)	0.39

D_2_O dilution technique	(*n* = 13)	(*n* = 9)	(*n* = 5)	Diff (95% CI)	*p*	Diff (95% CI)	*p*	Diff (95% CI)	*p*
Fat mass (kg)	3.1 ± 1.6	3.3 ± 1.3	2.4 ± 1.7	−0.14 (−1.7–1.4)	0.85	0.74 (−1.1–2.6)	0.40	0.88 (−1.1–2.8)	0.36
Fat‐free mass (kg)	18.4 ± 2.3	20.5 ± 3.7	20.5 ± 4.9	−2.16 (−5.1–0.81)	0.14	−2.1 (−5.9–1.6)	0.25	0.04 (−5.4–5.5)	0.99
Fat mass percentage (%)	14.6 ± 7.4	13.4 ± 4.2	11.0 ± 8.1	1.3 (−5.3–7.8)	0.69	3.6 (−5.2–12.4)	0.39	2.3 (−5.6–10.2)	0.53

*Note*: Values presented as mean ± SD. Summed Skinfold is four skinfold measurements combined (triceps, biceps, subscapular and suprailiac; the mean of left and right are used for the biceps and triceps skinfold). Differences tested with Linear mixed models. Adjusted for gender, birth weight SDS, age at visit and baseline value of outcome variable. Bold indicates statistical significance at *p* < 0.05.

Abbreviations: ADP, air‐displacement plethysmography; BF, breastfed; CTRL, control; FFMI, fat‐free mass index; FMI, fat mass index; mLP, modified low protein.

### 3.2 Body Composition

FM and FM% measured with ADP showed no significant differences between the two formula groups (Table 3; Figure S2A,C), while FFM was lower in the mLP formula‐fed group compared to the CTRL formula‐fed group after adjustments for confounders in the LMM analysis (difference −1240 g; 95% CI −1889 to −591; *p <* 0.001) (Table 3, Figure S2B). Both the mLP and CTRL formula‐fed groups had a significantly higher FM compared to the breastfed group (478 g, 95% CI: 108–847, *p* = 0.011; 472 g, 95% CI: 97.1–847, *p* = 0.014; respectively) (Table 3, Figure S2A). No significant differences were found in FMI and FFMI between the two formula groups or the breastfed reference group.

Body composition assessed using the D_2_O dilution technique showed no significant differences as well in FM and FFM between the formula groups (Table 3). Additionally, no significant differences were found between the breastfed reference group and the two formula groups. Although not statistically significant, a trend similar to that observed with body composition measured using ADP was noted, suggesting a lower mean FFM in the mLP formula‐fed group compared to the CTRL formula‐fed group (18.4 ± 2.3 vs. 20.5 ± 3.7, kg), and a lower FM in the breastfed reference group compared to both formula groups.

## 4 Discussion

This study found no differential growth pattern or body composition at the age of 6 years in children fed either the tested low protein infant formula with modified amino acid composition (containing 1.7 g protein/100 kcal) or a control infant formula (2.1 g protein/100 kcal) during the first 6 months of life. However, we observed that participants who received the mLP infant formula during the first 6 months of life had a lower fat‐free mass at the study visit at 6 years, compared to those who received a control infant formula. In contrast with our working hypothesis, this does not provide evidence that feeding the formula with reduced and modified protein content during early infancy would have a favourable impact on fat mass development in early childhood. However, the given sample size of 39 and 33 children, respectively, studied in the two randomised groups resulted in insufficient statistical power for detecting group differences of moderate size.

The results from the skinfold thickness technique and the deuterium oxide method for measuring body composition follow the same trend as the outcomes from the ADP method, showing no differences in body composition between the two formula groups. The only exception is the difference found with the ADP method, where FFM was lower in the mLP group compared to the CTRL group, although this was not replicated with the deuterium method. However, it is important to note that the sample size of children with a successful deuterium dilution measurement was very small, even smaller than the sample size for successful ADP measurements. Due to this limitation, our primary focus was on the ADP outcomes, with the deuterium dilution results being reported as additional data. The deuterium dilution method is particularly sensitive to measurement errors, especially in young children. Accurate measurements rely on the complete consumption of the labelled water by the child, and subsequent saliva samples must be correctly collected at home by the parents. Unfortunately, this process was not always executed properly, resulting in a very limited number of valid deuterium dilution measurements. Similarly, the skinfold thickness method, while less prone to the same issues, requires careful and consistent measurement across different sites on the body, which may have led to variability in the results. These factors highlight the difficulties associated with obtaining precise body composition data and underscore the importance of considering methodological limitations when interpreting the findings.

Growth measures up to the age of 2 years in children fed in infancy with the mLP and CTRL formulas were previously reported by us [25, 26]. At these ages we observed no differences in growth parameters or body composition between the mLP and CTRL formulas. However, when compared to the breastfed reference group, infants in both formula groups had a higher total body weight, with a higher fat mass and lower fat‐free mass at both 6 months and 2 years of age. At the age of 6 years, only a higher fat mass persisted, whereas other differences were not detected anymore. The current study is in line with previous trials and further underscores the potential increase in adiposity in infants who had received formula feeding compared with those who were breastfed [10, 13].

The composition of infant formulas has been a subject of ongoing debate, particularly regarding its differences with human milk and the potential modifications that can be made to optimise it [35, 36]. One particular interest has been the amount of protein in infant formula. On average, post‐transitional human milk contains approximately 1.0–1.3 g of protein per 100 mL, although this can vary depending on factors such as the stage of lactation and maternal BMI [37, 38]. It is well established that the protein concentration in breast milk gradually decreases as lactation progresses, whereas the protein content in infant formula remains constant over time. As a result, formula‐fed infants receive significantly higher amounts of protein during the first 6 months of life when compared to breastfed infants. The early protein hypothesis states that high early protein intake may stimulate the secretion of insulin and insulin‐like growth factor I, thereby enhancing weight gain in infancy [5]. Although the initial historic rationale for higher protein levels in infant formula was to accelerate infant weight gain and to safeguard essential amino acid requirements, more recent studies indicate that this accelerated weight gain velocity results in increased adipose tissue deposition alongside muscle mass, and in a large randomised trial also to an increased body fat percentage, which was linked to a higher risk of overweight or obesity during childhood [39, 40].

While planning the original RCT, we hypothesized that in addition to lowering the protein content, improving the amino acid composition would contribute to the expected positive outcomes, that is, optimisation of body composition development. However, the findings of this study do not provide evidence for lasting benefits of adopting the low‐protein formula approach with modified protein composition we tested here during the initial 6 months of life. Despite adapting the amino acid composition, lowering protein intakes with the protein composition used here could potentially even have had an unfavourable impact on body composition, based on the observed lower fat‐free mass compared to the control formula. However, one may question whether the observed mean difference of 1240 g in fat‐free mass is of clinical relevance when total fat free mass is approximately 20 kg. Furthermore, no differences were found in relative lean mass or in comparison to the breastfed reference group, suggesting that the mLP formula does not lead to poorer body composition outcomes.

In contrast, earlier RCTs examining the effects of high versus low protein content in infant formulas demonstrated significant beneficial changes in body composition. The CHOP trial (*n* = 1063) showed a difference in body composition in infants fed during the first 12 months of life infant formula with high protein (2.9 g/100 kcal, and follow‐on formula 4.4 g/100 kcal) versus an infant formula with a lower protein (1.8 g/100 kcal, and follow‐on formula 2.2 g/100 kcal) [17]. Infants fed the higher protein formula had a greater fat mass and greater fat‐free mass up to 6 years of age compared to those fed the lower protein formula [17]. Additionally, the higher protein group demonstrated higher BMI levels up to 11 years of follow‐up [41]. This study also included a breastfed reference group, which exhibited lower BMI trajectories up to 11 years compared to both formula‐fed groups [41]. In another RCT with a smaller sample size, the EPOCH study (*n* = 238), researchers found a lower weight gain rate in infants fed an infant formula with a relatively low protein content (1.8 g/100 kcal) compared to infants fed an infant formula with a high protein content (2.7 g/100 kcal) during the first months of life [18]. However, it is important to note that the high‐protein formulas used in both the CHOP and EPOCH studies contained protein levels which were substantially higher (2.9 and 2.7 g/100 kcal, respectively) than those in commercially available standard infant formulas, such as the ones used in our study (2.1 g/100 kcal versus 1.7 g/100 kcal). Consequently, the observed effects in the CHOP and EPOCH studies may have been more pronounced due to these higher protein intakes and suboptimal amino acid composition. The aim of our study was to determine whether reducing the protein content below the standard level, while optimising the amino acid profile, results in a more favourable body composition trajectory compared to infants fed a standard‐protein infant formula.

A meta‐analysis including 19 studies exploring the effects of feeding infant formula with different protein/energy ratios during the first 6 months of life, as well as full breastfeeding, on infant growth found evidence of faster weight gain in the first months of life with feeding formulas providing more than 2 g protein/100 kcal compared to those providing less protein, and compared to breastfeeding [42]. Also, a longer time period of feeding higher protein intakes (the first 12 compared to the first 6 months, as in our study) may contribute to greater effects than shorter feeding periods. However, it remains uncertain whether reducing the protein content further, below 2.1 g/100 kcal, would lead to growth and body composition trajectories more similar to infants fed human milk. The study by Ziegler et al., which examined a formula with 1.6 g protein/100 kcal for infants between 3 and 12 months, found a lower weight but no significant effects on body composition [43]. This study did, however, not include a long follow‐up period.

Although some studies have found significant differences in growth and body composition between infants fed with high‐protein and low‐protein formulas, reducing the protein content in infant formula is only one factor that can decrease the risk of childhood obesity [44, 45]. Avoiding overfeeding, excessive energy intakes, and feeds with a high glycemic load may contribute to preventing excessive infant weight gain. Other compositional differences between human milk and infant formula should also be considered to support a more similar growth trajectory and body composition as breastfed infants. It is important to acknowledge that not all differences can be replicated in infant formula. Unique elements, such as specific microbiota and the benefits of breastfeeding, including mother–child attachment, are intrinsic to human milk and breastfeeding, presenting challenges that may not be fully attainable through formula modification.

One of the many differences between breastmilk and infant formula that has received increasing attention over the past years is lipid quality. Lipids in human milk provide approximately half of the required energy for newborn infants [46]. The lipids in most infant formulas do not have a milk fat globule membrane like breastmilk does, so that the lipid droplets in formula are about 10 times smaller compared to those in breastmilk [47]. Since dietary lipid structure influences the way lipids are absorbed and how they are used for energy and storage, this might also partially explain the differences in growth and body composition development between formula‐fed and human milk‐fed infants [48, 49]. A recent study suggested that altering the formula lipid droplet structure, providing larger, milk‐phospholipid‐coated lipid droplets may support a lower BMI in early childhood as compared to feeding a standard formula [50, 51, 52].

For the present follow‐up study, a post hoc power analysis was conducted to assess the likelihood of detecting the observed differences in BMI among the three groups, revealing a power of only 37%. This relatively low power suggests that the ability to identify significant effects was likely hindered by participant attrition. Although the original intervention study was adequately powered, the decrease in the follow‐up population compromised the statistical strength of the results. To address this issue, we employed linear mixed models for our statistical analyses. This approach is particularly advantageous for handling longitudinal data, as it allows us to utilise information from participants who dropped out, thereby mitigating the impact of reduced power. While multiple imputation could also be considered, it does not enhance power and is often less stable than mixed models. Consequently, we advocate for the use of linear mixed models in our analysis to ensure more reliable conclusions.

Strengths of this study are the relatively long follow‐up period and a bicenter design that facilitates extrapolation of findings. We used four different methods to obtain information on growth and body composition: anthropometrics, skinfold thicknesses, ADP, and D_2_O dilution technique. The Bland–Altman analysis revealed no significant proportional bias between the measurement techniques, suggesting that the differences between ADP and D_2_O dilution do not systematically increase or decrease with changes in body composition. This implies that neither method consistently overestimates nor underestimates body composition relative to the other, supporting the reliability of both techniques across the measured range.

However, several limitations should be considered. The final sample sizes were small, with approximately 35 participants per group since our study experienced delays and high loss to follow‐up due to COVID‐19 pandemic restrictions in the community and hospitals. This resulted in a limited sample size, which may have contributed to both type 1 and type 2 errors, reflecting a lack of statistical power as mentioned earlier. Future studies should aim to minimise loss to follow‐up or increase the initial sample size to ensure sufficient power for detecting true effects in long‐term analyses. Also, the delay in executing the follow‐up resulted in the children being older than 6 years at the follow‐up visit, with the mean age of all children included being 6 years and 9 months, and a range of 35 months. Despite the age correction in the statistical analyses, the wide age range of participants may have influenced the ability to detect differences in growth pattern outcomes. Also, dietary intake and physical activity during childhood were not recorded, which may have influenced the outcomes in this study. The bicentric study design may potentially cause variability in the results between the centres. However, the inclusion of the study site as a covariate in our statistical analyses did not alter the observed differences, indicating that site‐specific variability did not significantly impact the results. Therefore, it was deemed unnecessary to include the centre as a covariate in our analysis. Furthermore, although each body composition measurement technique used in this study has its own inter‐variability [53], the results are relatively consistent with each other and thus strengthen our overall findings.

In conclusion, children who were fed the tested lower protein infant formula with modified amino acid composition during the first 6 months of life did not show a more beneficial growth pattern by age 6 compared to those fed standard formula. While significant differences in body composition were observed between the two formula groups and the breastfed group, the health implications of these differences remain unclear. Additionally, due to the limited sample size, these results should be interpreted with caution. Further research exploring other aspects of formula composition may help achieve growth and body composition patterns in formula‐fed infants that are more similar to those of breastfed infants.

## Author Contributions

J.B.G., S.M.P.K., B.K., and M.A.‐B. designed the research. J.M. and N.A. implemented and conducted the research. J.M. and J.W.R.T. performed the statistical analyses. D.H. conducted the lab analyses. J.M., B.J.K., C.H.P.A., and J.B.G. interpreted the data and wrote the paper. All authors critically revised the manuscript. J.M. has primary responsibility for the final content. All authors read and approved the final manuscript.

## Conflicts of Interest

S.M.P.K. reports receipt of speakers and consultancy honoraria from Nestlé Nutrition Institute and Nutricia; used as research funds. LMU and its employees N.A. and B.K. benefitted from support for scientific and educational activities from Barilla, Danone, DGC, DSM, Hipp, Nestlé, and reckitt. M.A.‐B. is an employee of Danone Research & Innovation. She had no role in the execution of the study or in the statistical analyses of the results. C.H.P.A. reports receipt of speakers and consultancy honoraria from Nestlé Nutrition Institute, Nutricia, and Baxter; used as research funds. J.B.G. is founder and director of the National Human Milk Bank, member of the National health Council and receives funding from RIVM to determine PFAS values in human milk.

## Supporting information


**Data S1.** Supporting Information.

## Data Availability

Data described in the manuscript, code book and analytic code will be made available upon request.
