# Supplementary material for: Anthropometry and Body Composition in Childhood: Follow‐Up of a Randomised, Double‐Blinded Controlled Trial With a Modified, Low‐Protein Infant Formula During Infancy
Source: Pediatr Obes. 2025 Jul 4;20(10):e70038. doi: 10.1111/ijpo.70038 (PMC12414579; doi:10.1111/ijpo.70038)

Anthropometry and body composition in childhood: follow-up of a randomized, double-blinded controlled trial with a modified, low-protein infant formula during infancy

Jacqueline Muts[1,2], Stefanie M.P. Kouwenhoven[3,4], Nadja Antl[5,6], Marieke Abrahamse-Berkeveld[7], Britt J. van Keulen[1,2], Jos W.R. Twisk[8], Dewi van Harskamp[9,10,11], Chris H.P. van den Akker[2,11,12], Berthold Koletzko[5,6], Johannes B. van Goudoever[1,2]

1. Department of Pediatrics, Emma Children's Hospital, Amsterdam UMC, University of Amsterdam, Amsterdam, The Netherlands.
2. Amsterdam Reproduction & Development research institute, Amsterdam UMC, Amsterdam, The Netherlands.
3. Erasmus MC-Sophia, Department of Pediatric & Neonatal Intensive Care, Rotterdam, The Netherlands.
4. Department of Internal Medicine, Division of Dietetics, Erasmus MC University Medical Centre Rotterdam, The Netherlands.
5. Department of Pediatrics, Dr. von Hauner Children's Hospital, LMU Hospital, LMU – Ludwig-Maximilians-Universität Munich, Munich, Germany.
6. German Center for Child and Adolescent Health, site Munich
7. Danone Research & Innovation, Utrecht, the Netherlands.
8. Department of Epidemiology and Data Science, Amsterdam University Medical center, Amsterdam, The Netherlands.
9. Amsterdam UMC, Department of Laboratory Medicine, Core Facility Metabolomics, Laboratory Genetic Metabolic Disease, University of Amsterdam, Amsterdam, Netherlands.
10. Amsterdam UMC, Emma Center for Personalized Medicine, Amsterdam, Netherlands.
11. Amsterdam UMC, Amsterdam Gastroenterology, Endocrinology & Metabolism research institute, Amsterdam UMC, Amsterdam, Netherlands.
12. Department of Neonatology, Emma Children's Hospital, Amsterdam UMC, University of Amsterdam, Amsterdam, The Netherlands.

Running title : ProtEUs 6year growth

Corresponding author

h.vangoudoever@amsterdamumc.nl

Emma Children's Hospital, Amsterdam UMC

Meibergdreef 9, 1105 AZ Amsterdam, The Netherlands

Supplementary Tables

Table 1. Amino acid composition of the study formulas

	**mLP formula**	**CTRL formula**
**Essential**	77/117	69/104
L-Isoleucine	102/155	143/217
L-Leucine	96/145	121/185
L-Methionine	28/41	31/46
L-Phenylalanine	43/65	56/85
L-Threonine	50/74	76/116
L-Trypthophan	12/19	76/36
L-Valine	80/122	74/112
**Nonessential**		
L-Histidine*	30/45	35/53
L-Alanine	24/37	42/63
L-Arginine	24/37	35/53
L-Aspartic acid/ L-Asparagine	101/153	132/200
L-Cyst(e)ine*	35/53	25/38
L-Glutamic acid/ L-Glutamine	202/306	284/432
Glycine	19/29	27/40
L-Proline	67/101	80/155
L-Serine	56/84	102/116
L-Tyrosine*	76/115	55/83

Values are Milligrams per 100 mL/per 100 kcal. mLP, modified low-protein; CTRL, control.
*Conditionally essential

Table 2. Patient characteristics of the original and follow-up cohort.

	Original cohort (n=245)	Follow-up cohort (n=106)
**Male (%)**	113 (46.1)	51 (47.2)
**Birth weight (kg)**	3.42 ± 0.37	3.40 ± 0.34
**Birth weight (SDS)**	0.26 ± 0.74	0.23 ± 0.70
**Caucasian (%)**	213 (87.3)	96 (88.9)
**Gestational age (weeks)**	39.7 ± 1.2	39.6 ± 1.1
**Maternal BMI at study entry (kg/m^2)**	25.7 ± 4.6	25.6 ± 4.5
**Primary end-point at 6 months, weight (kg)**	7.69 ± 0.83	7.70 ± 0.80
- **mLP**	7.74 ± 0.81	7.80 ± 0.90
- **CTRL**	7.90 ± 0.77	7.80 ± 0.65
- **BF**	7.34 ± 0.86	7.50 ± 0.77

Values presented as mean ± SD or n (%). BF, breastfed; CTRL, control; mLP, modified low protein.

Supplementary Figures

Figure 1 A-C. *Weight (A), Length (B), and BMI (C) at 6 years of age. Values represent interquartile ranges (IQR) with minimum and maximum bounds and compared by using linear mixed model analysis with adjustments for sex, birth-weight z-score, age at visit and baseline value. Dots outside the IQRs indicate outliers. mLP, modified low-protein formula; CTRL, control formula; BF, breastfed.*

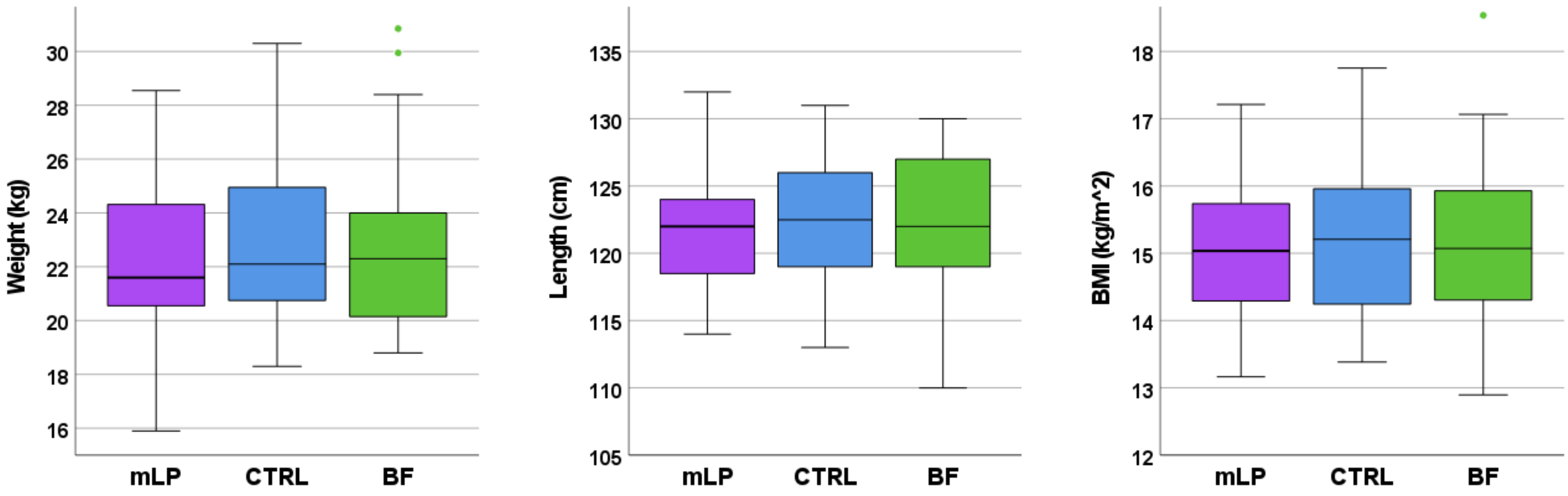

Figure 2 A-C. *Fat Mass (A), Fat-Free Mass (B), and Fat mass percentage (C) at 6 years of age measured with air-displacement plethysmography. Values represent interquartile ranges (IQR) with minimum and maximum bounds and compared by using linear mixed model analysis with adjustments for sex, birth-weight z-score, age at visit and baseline value. Dots outside the IQRs indicate outliers. *p < 0.05. mLP, modified low-protein formula; CTRL, control formula; BF, breastfed.*

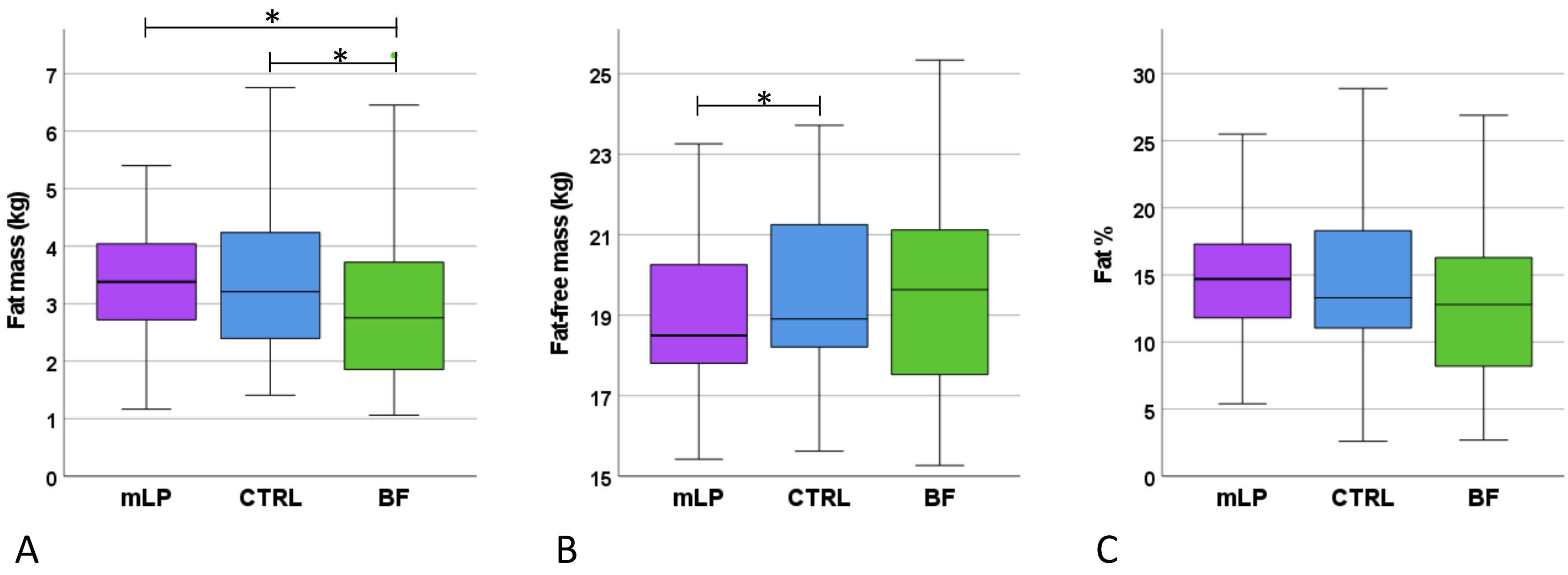

Supplement: Supplementary file 1 — Data S1. Supporting Information. [file IJPO-20-e70038-s001.pdf]
